# Torque-sensorless control of a high-ratio, backdrivable Wolfrom-gearbox for safe human-centered robotics

**DOI:** 10.3389/frobt.2026.1793978

**Published:** 2026-06-11

**Authors:** Léon Borremans, Stein Crispel, Tom Verstraten, Amin Khorasani, Greet Van de Perre, Pablo López Garcia

**Affiliations:** 1 Brubotics, Vrije Universiteit Brussel (VUB), Elsene, Belgium; 2 Flanders Make, Lommel, Belgium; 3 AILOS BV, Halle, Belgium; 4 Interuniversity Microelectronics Centre, Leuven, Belgium

**Keywords:** backdrivable, bandwidth, disturbance observer, double-inertia model, higher-order sliding-mode observer, high-ratio, ISO/TS 15066, Wolfrom

## Abstract

Safe physical human–robot interaction (pHRI) with compact, high-ratio actuators remains challenging in the absence of dedicated torque sensing. While high transmission ratios enable lightweight and efficient actuation, they are widely believed to degrade the ability to detect external disturbances through motor-side measurements — yet this limitation has not been rigorously quantified. We introduce a torque-sensorless control framework for a high-ratio, backdrivable Wolfrom gearbox (222:1), combining a double-inertia analytical model with a higher-order sliding-mode disturbance observer (HOSM-DOB). The analytical model yields a closed-form expression for disturbance-detection bandwidth as a function of gear ratio and motor inertia. The HOSM-DOB estimates external torques in real time using only motor-side measurements and is integrated into an impedance controller with active inertia shaping to reduce effective reflected inertia and mitigate impact forces. Experimental validation on a single-degree-of-freedom testbed, including controlled collisions measured with a Pilz Robot Measurement System (PRMS), demonstrates torque estimation errors below 10% for soft contacts. The HOSM-DOB outperforms a classical disturbance observer by 35% in detecting high-frequency impacts. Compliance with ISO/TS 15066 safety limits is confirmed up to a maximum motor speed of 7000 rpm, comparable to existing collaborative robot platforms. The bandwidth analysis reveals a fundamental trade-off between gear ratio, motor inertia, and sensing performance, demonstrating that classical inertia-matching principles do not maximize impact detectability. The results establish that high-ratio, backdrivable actuators — when combined with observer-based control — can achieve both high torque density and safe interaction, providing a viable alternative to direct-drive and harmonic-drive solutions in human-centered robotics.

## 1 Introduction

### 1.1 State-of-the-art

In physical human-robot interaction (pHRI) applications, such as collaborative manufacturing, contact between humans and robots might occur at any moment in time. That means that safety is crucial for the deployment of robots in this field. Until now, safety has been achieved by combining two approaches. On the one hand, the operating speed of the actuators is reduced (Lopez Garcia et al., 2020). This ultimately leads to slower, and thus less productive manipulation and/or collaboration. On the other hand, some actuators come with integrated torque sensors^1^. The measurements of such a sensor can be used for the estimation of the external torque acting on the output of the actuator. However, these sensors increase cost, size, mass and complexity of the robot, making them potentially less safe (Matsuki et al., 2019).

In cobots two primary actuator architectures are commonly considered: quasi-direct-drive actuators, which employ a large motor coupled with a low-ratio transmission, and harmonic-drive actuators, which use a smaller motor in combination with a mid-ratio gear mechanism (Lopez Garcia et al., 2020). Quasi-direct-drive actuators offer the significant advantage of being backdrivable, enabling high-bandwidth response and reliable impact detection without dedicated torque sensors (Wensing et al., 2017). However, they tend to be relatively large and heavy. In contrast, harmonic-drive actuators are more compact and lightweight but are typically non-backdrivable, necessitating the use of torque sensors for accurate torque estimation and control (Kazerooni, 1995).

To overcome the limitations associated with poor backdrivability, a high-ratio, high-efficiency Wolfrom-type gearbox with a transmission ratio of 222:1 was developed at Vrije Universiteit Brussel (Crispel et al., 2021). This gearbox enables the use of a compact motor (Lopez Garcia et al., 2022a) while remaining backdrivable^1^, resulting in a lightweight and inherently safe actuator, even in an unpowered state. In this context, *backdrivability* is defined as the capability to detect load-side torques at the motor input when power flows from the load to the actuator. Owing to this property, the Wolfrom-based actuator is well suited for cobots, humanoid robots, and exoskeletons, where low mass and intrinsic backdrivability are essential for human safety and device wearability.

To the best of the authors’ knowledge, it is widely accepted that high gear ratios reduce an actuator’s intrinsic bandwidth of sensing disturbance torques via motor-side measurements. However, no explicit mathematical formulation supporting this claim has been reported in the literature (Lopez Garcia et al., 2022a; Du et al., 2021; Roos and Wikander, 2006). In contrast, Roos et al. argue that increasing the gear ratio improves the bandwidth of the transfer function relating motor torque to motor acceleration, which is beneficial for control design purposes (Roos and Wikander, 2006). Nevertheless, the system behavior under (impact) disturbance conditions—specifically, the bandwidth of the transfer function relating disturbance torque to motor acceleration—is not addressed. This bandwidth provides a measure of an actuator’s ability to sense load-side disturbances using motor-side measurements, a capability that is critical for robotic applications that do not employ torque sensors.

ISO/TS 15066 specifies the maximum permissible robot speeds and contact forces or pressures to ensure safe human–robot collaboration. These limits depend on the impacted body region and are derived from an impact energy model that assumes a worst-case, fully inelastic collision. The model incorporates effective stiffness and mass parameters for both the human and the robot to determine allowable speed thresholds that prevent excessive impact forces or pressures (International Organization for Standardization, 2016). Human–robot contacts are classified as either transient or quasi-static. *Transient contacts* correspond to impacts where separation occurs immediately after contact, whereas *quasi-static contacts* involve sustained clamping or crushing. The allowable force and pressure limits for transient contacts are typically twice those for quasi-static contacts, but they may only be applied for durations up to 0.5 s , while quasi-static limits may be applied indefinitely (International Organization for Standardization, 2016).

### 1.2 Author’s contribution and outline

**FIGURE 1:**
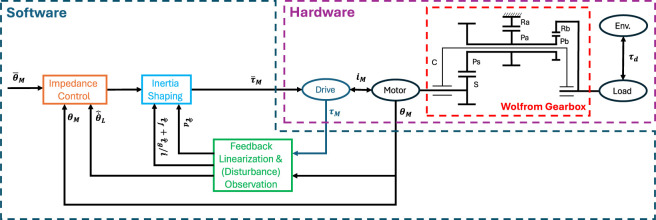
Overview of the control architecture (software) and actuator system (hardware). A motor-drive unit drives the load through a high-ratio Wolfrom gearbox, while a HOSM-DOB estimates external torques from motor-side signals and feeds an impedance controller with active inertia shaping.

This work addresses the challenge of achieving safe physical human–robot interaction with compact, high-ratio actuators in the absence of dedicated torque sensing by developing a control framework for high-ratio, backdrivable Wolfrom actuators. A high-level overview of the proposed architecture is shown in Figure 1. The diagram illustrates the interaction between the motor-side dynamics, the Wolfrom gearbox, and the load, as well as the coupling with the environment. It further highlights the integration of a higher-order sliding-mode disturbance observer (HOSM-DOB) within an impedance control framework. The estimated disturbance torque is used for active inertia shaping, enabling improved impact detection and mitigation without dedicated torque sensing.

The main contributions of this paper are twofold. First, this work provides a rigorous mathematical proof of the widely accepted assumption that high-ratio actuators impose bandwidth limitations on impact detection when relying solely on motor-side measurements (Lopez Garcia et al., 2022a; Du et al., 2021). In doing so, the analysis extends the results of Roos et al., which are limited to the effect of gear ratio on the bandwidth of actuator response to motor-side torque inputs.

Second, a disturbance observer based on higher-order sliding-mode theory is developed, specifically tailored to torque-sensorless, high-ratio actuators. Compared to classical disturbance observers for linear systems, the proposed observer enables faster and more accurate estimation of environmental torques, making it well suited for applications requiring rapid impact detection and compliant control.

The remainder of this paper is organized as follows. Section 2 provides background on the ISO/TS 15066 standard and discusses its limitations. Sections 3–5 present the main contributions of this work. Section 3 derives the kinematic and dynamic models of the Wolfrom-based actuator and develops the bandwidth analysis and disturbance observer. Section 4 introduces the control framework, and Section 5 presents the simulation and experimental validation.

## 2 ISO/TS 15066: human-robot collision behavior

In this section, the ISO/TS norm concerning robot-human collisions is reproduced. Every robot must satisfy this norm in order to be safely deployed in a pHRI environment. The norm will provide a threshold for the impact force value that the robot cannot exceed for a given impacted human body part.

The ISO/TS 15066 norm models the collision between robot and human as an impact between two masses. It adopts a conservative assumption in which the human absorbs the full post-impact energy (Fischer et al., 2023). In this formulation, the reduced mass between the human and robot is given by:

$$\mu ={\left(\frac{1}{m_{H}}+\frac{1}{m_{R}}\right)}^{-1},$$ (1)

where $m_{H}$ and $m_{R}$ denote the human and robot mass, respectively. The latter can be calculated as:

$$m_{R}=\frac{M}{2}+m_{L},$$ (2)

where M is the total mass of all moving parts of the robot and $m_{L}$ is the payload mass. Based on the reduced mass a maximum impact energy is formulated as:

$$E_{\text{max}}=\frac{F_{\text{max}}^{2}}{2K}=\frac{A_{c}^{2}p_{\text{max}}^{2}}{2K}=\frac{1}{2}\mu v_{\text{rel,max}}^{2},$$ (3)

with $F_{\max}$ , $p_{\max}$ , K and $A_{c}$ the maximum allowable force, pressure thresholds, effective stiffness and surface area, which all depend on the body region of impact, provided in tables in (International Organization for Standardization, 2016). Equation 3 can be used to determine the maximum allowable relative speed $v_{\mathrm{rel,max}}$ between human and robot. The idea is that, when this speed threshold is not exceeded, a possible impact will not result in exceeding the force threshold (International Organization for Standardization, 2016).

There are, however, some limitations regarding this formula that are relevant to highlight. On one hand, it does not take into account the possibility of a ‘safe’ control design, which can in fact lower the impact force with fast response (Khorasani et al., 2024). On the other hand, the calculation of the robot mass $m_{R}$ is independent of the robot configuration at the moment of impact. However, it is experimentally proven in (Kirschner et al., 2021) that the effective mass of the robot heavily depends on the configuration at the moment of impact. Khatib et al. provides a formula to calculate a better approximation for the robot effective mass $m_{R}$ (Khatib, 1995).

## 3 High-ratio Wolfrom-based actuator layout with observer-based torque estimation

In this section, a complete mathematical model of the Wolfrom-based actuator is derived. From that model, the problem of high gear ratio onto the bandwidth of sensing disturbance torques is proven. Subsequently, due to the absence of the torque sensor, a disturbance observer is derived for fast real-time estimation of the disturbance torque. In the next section, Section 4, it is explained how this signal is used to shape the inertia of the actuator.

### 3.1 Wolfrom-based gearbox kinematics and dynamics

**FIGURE 2:**
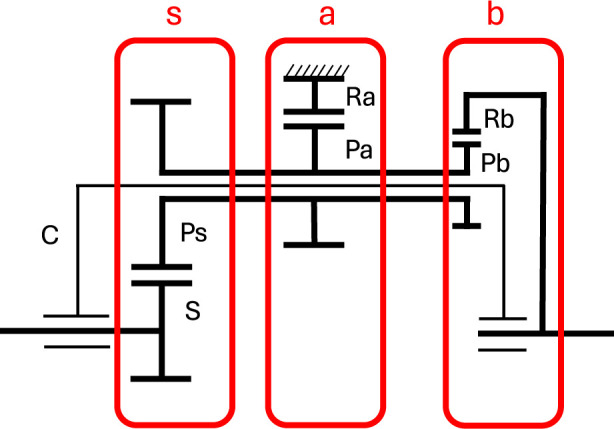
Schematics of the Wolfrom gearbox with pre-stage s. Sun S is connected to the input (motor side) and ring Rb to the output (load side). The amount of identical planets are two, two and four for Ps, Pa and Pb respectively. The total gear ratio is i=222 .

A robotic actuator consists of an electric motor and a gearing element that amplifies the torque delivered to the joint. The gearbox layout in this paper is depicted in Figure 2 and is extensively explained in (Crispel et al., 2021; Lopez Garcia et al., 2022b). When the motor drive allows 4-quadrant operation, the current signal can be exploited in the control architecture, for instance, for the detection of impacts (Haddadin et al., 2017).

In line with (Du et al., 2021), the dynamic model of the actuator for a single-degree-of-freedom robot arm is described by:

$$\tau_{M}=J_{M}{\ddot{\theta }}_{M}+\tau_{f}+\frac{\tau_{s}}{i}$$ (4a)

$$\tau_{s}=J_{L}{\ddot{\theta }}_{L}+\tau_{g}+\tau_{d}$$ (4b)

$$\tau_{s}=K_{s}\left(\frac{\theta_{M}}{i}-\theta_{L}\right),$$ (4c)

where $J_{M}$ and $J_{L}$ denote the motor-side and load-side inertia’s of the complete actuator, respectively. The angular positions on the motor and load sides are represented by $\theta_{M}$ and $\theta_{L}$ , with a gear ratio i relating both sides. (^..^) over a variable indicates its first (second)-order time derivative. The torques $\tau_{M}$ , $\tau_{s}$ , $\tau_{f}$ , $\tau_{g}$ , and $\tau_{d}$ correspond to the motor torque, the load-side reflected reaction torque, the total friction torque, the gravity-induced torque, and an external disturbance torque, respectively. From Equation 4b it can be seen that the reaction torque $\tau_{s}$ is the sum of all torques acting on the load side and can be directly measured when a torque sensor is placed at the load side of the gearbox. The motor torque $\tau_{M}$ is typically modeled as the product of the motor torque constant and the total drawn current (Bejarano et al., 2007). The parameter $K_{s}$ represents the total actuator stiffness reflected to the load side.

The proposed model assumes that all losses, including gearbox losses, are lumped in the friction torque. This assumption is only valid when the gearbox has a high efficiency (Verstraten et al., 2015).

Although gravity torques $\tau_{g}$ are often included within the disturbance torque $\tau_{d}$ , they are modeled separately here in order to explicitly isolate the contribution of nominal gravitational loads acting on the joint.

The motor- and load-side inertia’s can be expressed as:

$$J_{M}=J_{\text{motor}}+J_{\text{motor}-\text{flange}},$$ (5a)

$$J_{L}=J_{G}+J_{\text{load}-\text{flange}}+J_{\text{robot}},$$ (5b)

where each term represents the inertia contribution of the corresponding mechanical component. $J_{G}$ is the load-side reflected gearbox inertia.

In general, gearbox efficiency and friction characteristics depend on both transmitted torque and rotational speed (Del Castillo, 2002; Pennestri and Freudenstein, 1993), leading to complex and nonlinear friction behavior. However, due to the relatively high efficiency of the considered gearbox compared to harmonic drive actuators (Lopez Garcia et al., 2020), we adopt a simplified Coulomb–viscous friction model:

$$\tau_{f}=\nu {\dot{\theta }}_{M}+\tau_{c}\text{ sign}\left({\dot{\theta }}_{M}\right).$$ (6)

This model captures the dominant friction effects while keeping the formulation simple. It is a reasonable approximation because the actuator exhibits a nearly constant efficiency profile, making small variations in friction negligible for the purposes of observer and control design.

In order to find an expression for the gearbox inertia $J_{G}$ , the Willis equations are written for the gearbox schematics depicted in Figure 2:

$$\frac{\omega_{S}-\omega_{C}}{\omega_{Ps}-\omega_{C}}=-\frac{Z_{Ps}}{Z_{S}},$$ (7a)

$$\frac{\omega_{Ps}-\omega_{C}}{\omega_{Ra}-\omega_{C}}=+\frac{Z_{Ra}}{Z_{Pa}},$$ (7b)

$$\frac{\omega_{Pb}-\omega_{C}}{\omega_{Rb}-\omega_{C}}=+\frac{Z_{Rb}}{Z_{Pb}},$$ (7c)

with $\omega_{j}$ and $Z_{j}$ the rotational speed and number of teeth of component j , respectively. The three planets Ps , Pa and Pb are rigidly attached to each other and thus their speeds are equal: $\omega_{Ps}$ = $\omega_{Pa}$ = $\omega_{Pb}$ ≔ $\omega_{P}$ . In order to use the concept of reflected inertia, Equations 7a–c must be solved to the ratios $\frac{\omega_{C}}{\omega_{Rb}}$ , $\frac{\omega_{P}}{\omega_{Rb}}$ and $\frac{\omega_{S}}{\omega_{Rb}}$ , that relate the corresponding component rotational speeds to the output speed $\omega_{Rb}$ . These ratios can then be used in the formula for reflected inertia, again assuming 100% gearbox efficiency:

$$J_{G}=\underset{j}{\sum }{\left(\frac{\omega_{j}}{\omega_{Rb}}\right)}^{2}J_{j},$$ (8)

with $J_{j}$ the mass inertia of component j , which can be estimated from CAD files. Since ring Ra is stationary, its speed is zero: $\omega_{Ra}=0$ . This results in a formula for the gearbox inertia:

$$J_{G}=i_{0}^{2}J_{C}+{\left(i_{a}i_{w}\right)}^{2}\left(2J_{Ps}+4\left(J_{Pa}+P_{Pb}\right)\right)+i^{2}J_{S}+J_{Rb},$$ (9)

with separate gear ratios as defined in (Crispel et al., 2021):

$$i_{a}\overset{\Delta }{=}1-\frac{Z_{Ra}}{Z_{Pa}},$$ (10a)

$$i_{0}\overset{\Delta }{=}\frac{\omega_{S}}{\omega_{C}}=1+\frac{Z_{Ra}Z_{Ps}}{Z_{Pa}Z_{S}},$$ (10b)

$$i_{w}^{-1}\overset{\Delta }{=}\frac{\omega_{Rb}}{\omega_{C}}=1-\frac{Z_{Pb}Z_{Ra}}{Z_{Pa}Z_{Rb}},$$ (10c)

$$i\overset{\Delta }{=}\frac{\omega_{S}}{\omega_{Rb}}=i_{0}i_{w}.$$ (10d)

Compared to (Crispel et al., 2021), $i_{a}$ is a newly defined gearbox inertia to simplify the equations.

### 3.2 Bandwidth reduction problem for the detection of impacts

In this section, we derive a mathematical expression for the bandwidth of the transfer function relating disturbance torque to motor acceleration. We refer to this transfer function as the *backward drive transfer* B(s) . The bandwidth of the backward drive transfer is interpreted as the maximum disturbance frequency that can be detected by a motor-side encoder, a metric of particular importance for intrinsically safe, sensorless actuators. In contrast, following (Roos and Wikander, 2006), we define the *forward drive transfer* F(s) of an actuator as the transfer function relating motor torque to motor acceleration. This metric is commonly used to quantify actuator performance when a motor-side encoder is employed (Roos and Wikander, 2006). By deriving the transfer functions corresponding to both forward and backward drive, we can identify what influence parameters like gear ratio and motor inertia have on the dynamic response and safety of an actuator.

Consider for this the double-inertia model previously described in Equations 4a–c without friction and gravity torques. The resulting system becomes linear and, after taking the Laplace transform, can be solved to the motor side acceleration:

$${\ddot{\theta }}_{M}\left(s\right)=F\;\left(s\right)\tau_{M}\left(s\right)-B\;\left(s\right)\frac{\tau_{d}\left(s\right)}{i},$$ (11)

where the negative sign in front of the backward drive transfer reflects backdriving behavior: for a positive disturbance torque and zero motor torque, the actuator experiences a negative motor acceleration. The solution for the forward F(s) and backward drive transfers B(s) are:

$$F\left(s\right)\;=\frac{i^{2}\left(J_{L}s^{2}+K\right)}{i^{2}J_{M}J_{L}s^{2}+K\left(i^{2}J_{M}+J_{L}\right)}$$ (12a)

$$B\left(s\right)\;=\frac{i^{2}K}{i^{2}J_{M}J_{L}s^{2}+K\left(i^{2}J_{M}+J_{L}\right)}.$$ (12b)

The bandwidth of these transfer functions are defined as the angular frequency at which the magnitude has decreased by 3 dB . Accordingly, the bandwidth $\omega_{BW}$ for the forward and backward drive transfers can be obtained by solving:

$${\left|G\left(s\right)\right|}_{s=j\omega_{BW}}≔\frac{1}{\sqrt{2}}{\left|G\left(s\right)\right|}_{s=0},$$ (13)

after substituting G(s) for F(s) or B(s) . In order to better highlight the influence of motor inertia for a given load inertia, introduce the inertia ratio as $\alpha^{2}≔J_{L}/J_{M}$ . In doing so, the following expression for the bandwidth of an actuator under disturbance torques can de derived:

$$\omega_{BW}^{2}=C\;\left(1+\frac{\alpha^{2}}{i^{2}}\right),$$ (14a)

$$C=\frac{\left(1+\sqrt{2}\right)K_{s}}{J_{L}},$$ (14b)

which is an estimate for the solution of Equation 13 after substituting G(s) for B(s) . This formula can also be written in terms of its dynamic parameters:

$$\omega_{BW}^{2}=\left(1+\sqrt{2}\right)K_{s}\left(\frac{1}{J_{L}}+\frac{1}{i^{2}J_{M}}\right).$$ (15)

**FIGURE 3:**
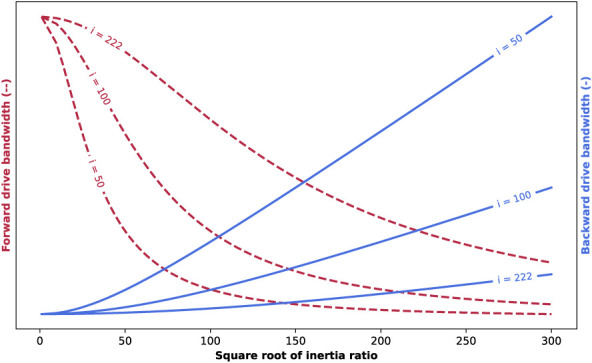
Influence of gear ratio (i) and inertia ratio $\left(\alpha^{2}=J_{L}/J_{m}\right)$ on actuator bandwidth (forward: red dashed, backward: blue solid). Parameters: $K_{s}=10^{4}\;Nm$ (Liu et al., 2018), $J_{L}=1\;kg.m^{2}$ . Increasing α reduces forward but increases backward bandwidth, while increasing i shows the opposite trend.

Equation 14a and the solution of the bandwidth for the forward drive are illustrated in Figure 3. It shows that the inertia-matching principle is not applicable when the objective is to maximize the bandwidth of backward drive. The inertia-matching principle, originally introduced in (Pasch and Seering, 1984), states that maximum load-side acceleration for a unit motor-side torque is achieved when the gear ratio is chosen as the square root of the inertia ratio, i.e., i=α .

Figure 3 illustrates the multiple trade-offs involved in actuator design. The bandwidth of the forward drive increases with higher gear ratios and lower inertia ratios, whereas the bandwidth of the backward drive increases with lower gear ratios and higher inertia ratios. In practice, however, gear ratio and inertia ratio are positively correlated, since achieving a larger reduction ratio typically requires a smaller motor and thus lower motor inertia. Consequently, enhancing forward drive bandwidth comes at the expense of backward drive bandwidth, and vice versa. Moreover, the maximum forward or backward drive bandwidth cannot be attained independently due to this practical correlation between gear ratio and inertia ratio.

Figure 3 can therefore serve as a guideline for selecting the gear ratio during actuator design. For example, given a specific motor and robotic arm, the gear ratio can be chosen to balance the forward and backward drive bandwidths. This is possible because, as shown in Equation 11, the forward and backward drives share the same sign, order of magnitude (both reflected to the motor side), and units, enabling direct comparison for design optimization.

This qualitative study shows that an actuator simultaneously achieving high dynamic performance and good impact detectability necessarily involves a trade-off between gear ratio and inertia ratio.

In general, actuator stiffness is influenced by both the gear ratio and the inertia ratio (Roos and Wikander, 2006); however, as shown in Equation 15, the bandwidth increases monotonically with stiffness. Therefore, a more detailed analysis of stiffness is not required in this qualitative context.

### 3.3 Torque-sensorless disturbance observer

In this work, a higher-order sliding-mode disturbance observer is derived to cope with the absence of a torque sensor. For stability analysis, it is assumed that the gearbox exhibits sufficiently high stiffness such that the control scheme can rely solely on motor-side measurements. Under this assumption, a load-side encoder is not required and the double-inertia model, Equations 4a–c, can be simplified to

$$J{\ddot{\theta }}_{L}+i\tau_{f}+\tau_{g}+\tau_{d}=i\tau_{M},$$ (16)

where J is the total reflected inertia at load side in case of 100 % gearbox efficiency:

$$J=J_{L}+i^{2}J_{M}.$$ (17)

In the presence of gearbox efficiency η , the reflected inertia becomes $J=\frac{J_{L}}{\eta }+i^{2}J_{M}$ (Verstraten et al., 2015). The sensitivity of the total reflected inertia to variations in efficiency can be expressed as $S_{\eta }≔\frac{\eta }{J}\frac{\partial J}{\partial \eta }=\frac{-J_{L}}{\eta i^{2}J_{M}+J_{L}}\overset{i\to \infty }{\to }0$ . Due to the typically high efficiency η and large gear ratio i , this sensitivity remains small. Therefore, assuming a constant high efficiency is justified when calculating the total reflected inertia. In the experimental validation section, the parameters of the Wolfrom-based actuator are given in Table 2. Using these values with a gearbox efficiency of 85% , the resulting sensitivity is approximately 2.5% , confirming that the effect is small. In comparison, for a gear ratio of 10 instead of 222, the sensitivity would be 91.0% .

Equation 16 can be used to estimate the disturbance torque:

$${\hat{\tau }}_{d}=i\tau_{M}-{\hat{\tau }}_{f}-{\hat{\tau }}_{g}-\hat{J}{\hat{\ddot{\theta }}}_{L},$$ (18)

where the ˆ signifies an estimation of that quantity. This method heavily depends on how well the friction ${\hat{\tau }}_{f}$ and gravity ${\hat{\tau }}_{g}$ models represent the real friction and gravity contributions (Khorasani et al., 2024). In addition, double differentiation of the position (i.e., measured with an encoder) induces extra noise, thus low bandwidth filtering is needed (Tilli and Montanari, 2001), leading to a higher risk of instability.

A solution is the use of a Luenberger observer in combination with a higher-order sliding-mode differentiator, as theoretically explained in (Bejarano et al., 2007). Applied to this actuator: when the input signal is chosen as $u=\tau_{M}-{\hat{\tau }}_{f}-\frac{{\hat{\tau }}_{g}}{i}$ , system Equation 16 becomes linear and can be written in the state space form $\dot{x}=Ax+Bu+D\tau_{d}$ , with states $x={\left[\begin{array}{c} \theta_{L}\;{\dot{\theta }}_{L} \end{array}\right]}^{T}$ and system matrices $A=\left[\begin{array}{cc} 0 & 1\\ 0 & 0 \end{array}\right]$ , $B={\left[\begin{array}{c} 0\;\frac{i}{J} \end{array}\right]}^{T}$ , $C=\left[\begin{array}{c} 1\;0 \end{array}\right]$ and $D={\left[\begin{array}{c} 0-\frac{1}{J} \end{array}\right]}^{T}$ . The Luenberger observer estimates the states as $\dot{z}=Az+\hat{B}u+L\left(\theta_{L}-Cz\right)$ , with $\hat{B}={\left[\begin{array}{cc} 0 & i/\hat{J} \end{array}\right]}^{T}$ and $L={\left[\begin{array}{cc} l_{1} & l_{2} \end{array}\right]}^{T}$ . Due to the disturbance $\tau_{d}$ , z will not reach x . This can be solved by introducing a signal v that is updated according to the difference between the actual $\left(\theta_{L}\right)$ and estimated load position (Cz) :

$${\dot{v}}_{1}=-\alpha_{3}N^{1/3}{\left|v_{1}-\left(\theta_{L}-Cz\right)\right|}^{2/3}\text{sign}\left(v_{1}-\left(\theta_{L}-Cz\right)\right)+v_{2},$$ (19a)

$${\dot{v}}_{2}=-\alpha_{2}N^{1/2}\left|v_{2}-{\dot{v}}_{1}\right|\text{sign}\left(v_{2}-{\dot{v}}_{1}\right)+v_{3},$$ (19b)

$${\dot{v}}_{3}=-\alpha_{1}N\text{ sign}\left(v_{3}-{\dot{v}}_{2}\right),$$ (19c)

with N a hyper parameter that must suit the expression $N>|CAD\|\tau_{d}^{\max}|$ , with $\tau_{d}^{\max}$ the maximum expected disturbance (Bejarano et al., 2007). According to the theory described in (Bejarano et al., 2007) and after some manipulations, the estimated disturbance can be expressed as:

$${\hat{\tau }}_{d}=-\hat{J}\;\left(v_{3}+l_{2}v_{1}+l_{1}v_{2}\right).$$ (20)

Bejarano et al., (2007) also explains a method to update the measurements of the improved states x , which is needed in the friction model. Equations 19a–c, Equation 20 shows that only the position measurement is required to observe the disturbance torque, making this method faster in estimation compared to Equation 18. A drawback, however, is the higher computational cost when implemented in real time, which should be considered when implementing the proposed methodology on specific target hardware to guarantee real time performance and stability.

For computational analysis, simulated data is used to assess the computational burden of both observers. A total of 50 runs are performed and averaged, using a backward Euler discretization with a sampling time of 1 ms over a simulation horizon of 5 s . The mean computation time of the simple DOB is 43 μs , while the HOSM-DOB requires 640 μs . This can be reduced to 510 μs , corresponding to an improvement of approximately 25% , by minimizing arithmetic operations through pre-computation of constants and exploiting the identity $|x{|}^{n}sign\;\left(x\right)=x|x{|}^{n-1}$ . Overall, the HOSM-DOB is approximately 15 times more computationally demanding than the simple DOB. Nevertheless, the absolute execution time remains well below the sampling period of 1 ms . Hence, despite the increased computational complexity, the execution time remains on the order of $10^{-4}$ to $10^{-5}\;s$ per evaluation, confirming that the proposed observer is suitable for real-time implementation and that the additional computational cost is justified by the improved estimation performance.

## 4 Control system design for the Wolfrom-based gearbox for the deployment in a safe pHRI environment

In this section, an impedance control scheme utilizing the higher-order sliding-mode disturbance observer (HOSM DOB) is proposed. With this disturbance observer the total effective inertia of Equation 17 and thus of the motor can be set by the programmer, mitigating impact forces (Khorasani et al., 2024).

**FIGURE 4:**
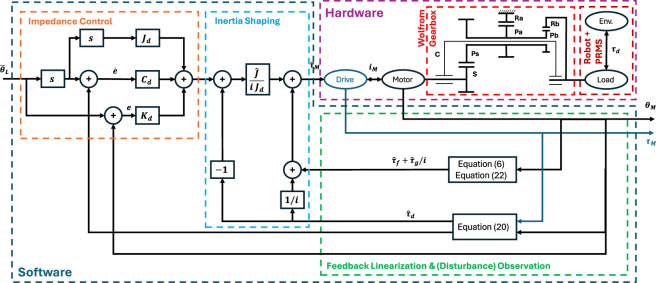
Schematics of the impedance control for safe pHRI, without torque feedback and with disturbance observer. The disturbance observer is used to estimate the disturbance torque $\tau_{d}$ and speed ${\dot{\theta }}_{L}$ in real time. These signals are used in an impedance control scheme to achieve safe pHRI.

Impedance control is often used to shape the dynamic response of a system under external forces (Khorasani et al., 2024; Siciliano et al., 2025). When impedance control is included with torque and/or force feedback or with a disturbance observer, the reflected inertia can be artificially shaped to a virtual inertia $J_{d}$ (Siciliano et al., 2025). Consider for this the simplified model of Equation 16 and set the reference torque equal to:

$${\bar{\tau }}_{M}≔\frac{\hat{J}}{iJ_{d}}\left(J_{d}{\ddot{\bar{\theta }}}_{L}+C_{d}\dot{e}+K_{d}e-{\hat{\tau }}_{d}\right)+{\hat{\tau }}_{f}+\frac{{\hat{\tau }}_{g}+{\hat{\tau }}_{d}}{i},$$ (21)

with error signal $e={\bar{\theta }}_{L}-\theta_{L}$ , ${\bar{\theta }}_{L}$ the reference signal in time and $C_{d}$ , $K_{d}$ the virtual damping and stiffness respectively. The estimated disturbance ${\hat{\tau }}_{d}$ is the signal computed with Equation 20 when the HOSM DOB is used and Equation 18 when the Simple DOB is used. The estimated friction model ${\hat{\tau }}_{f}$ is computed with Equation 6 and the estimated gravity torque ${\hat{\tau }}_{g}$ is:

$${\hat{\tau }}_{g}=G\;\sin\left(\theta_{L}\right),$$ (22)

with G the torque weight of the link at a load angle of $\theta_{L}=90{}^{\circ}$ . When the electric time constant of the motor is considered small $\left({\bar{\tau }}_{M}\approx \tau_{M}\right)$ and perfect model knowledge is assumed ( ${\hat{\tau }}_{f}\approx \tau_{f}$ , ${\hat{\tau }}_{g}\approx \tau_{g}$ , ${\hat{\tau }}_{d}\approx \tau_{d}$ and $\hat{J}\approx J$ ), inserting Equation 21 into the dynamics of Equation 16 results in:

$$\tau_{d}=J_{d}\ddot{e}+C_{d}\dot{e}+K_{d}e,$$ (23)

which is stable $\forall \;J_{d},C_{d},K_{d}>0$ if $|\tau_{d}\left(t\right)|<+\infty \;\forall t$ (Nicotra et al., 2015). In order to shape Equation 23 the parameters $J_{d}$ , $C_{d}$ , and $K_{d}$ can be chosen independently, allowing one to modify the reflected inertia of the system from J to $J_{d}$ . The complete control architecture is provided in Figure 4.

## 5 Experimental setup, simulations and validation

In this section the experimental setup is described, which is used to simulate a single-degree-of-freedom robot colliding with a human body part in accordance with ISO/TS 15066. The setup is also implemented in a Matlab/Simulink/Simscape environment, hereafter referred to as the virtual twin (VT). Both the physical setup and the virtual twin are controlled using the strategy of Equation 21, as shown in Figure 4. The control and virtual twin parameters are tuned through an open-loop experiment. The resulting impact measurements are then compared with those from the virtual twin to validate the correspondence between experimental and simulated behavior. Finally, two additional experiments assess the ability of the Wolfrom-based gearbox to comply with the ISO/TS 15066 requirements for safe pHRI.

### 5.1 Hardware and virtual twin setup

**FIGURE 5:**
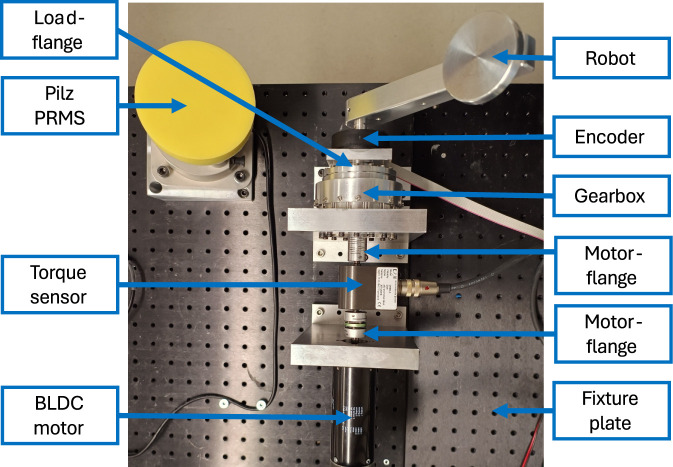
Experimental setup for the simulation of human-robot impacts. The PRMS device is used to simulate different human body parts in accordance with the ISO/TS 15066 norm.

**FIGURE 6:**
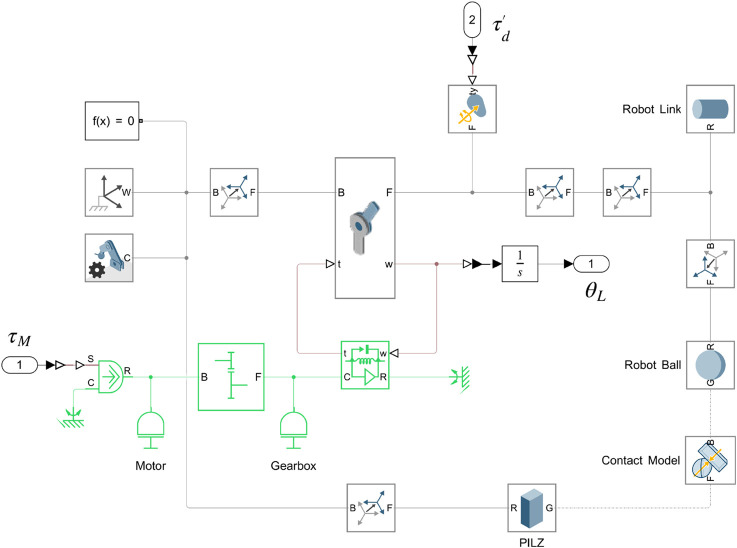
Virtual twin (VT) of the experimental setup in Figure 5, implemented in Matlab/Simulink/Simscape and used as the ‘Wolfrom gearbox’ and ‘Robot + PRMS’ in Figure 4. $\tau_{d}^{\prime }$ denotes disturbance torque without impact. The ‘Contact Model’ simulates impact between the robot endpoint and the PILZ, modeled as a stiff, damped plate (Table 1).

This section describes the hardware setup, which is illustrated in Figure 5. The Pilz Robot Measurement System (PRMS) is used to simulate a collision between robot and human. The PRMS^2^ is a commercial device capable of measuring the force/pressure exerted on it at a sampling frequency of 2 kHz . Different springs and contact pads^3^ are available to mimic different human body parts, as described in Table 1. A position $\theta_{L}=0$ corresponds to the link (‘robot’) being in a vertical, downward position. The chosen brush-less DC (BLDC) motor is a 24V EC45 250 W Maxon motor controlled in torque-mode with EtherCAT (a real-time Ethernet-based fieldbus protocol) at a sampling frequency of 1 kHz (Langlois et al., 2018). The virtual twin of the hardware setup is constructed in Matlab/Simulink and is presented in Figure 6.

**TABLE 1:** PRMS human equivalent spring, damping and max biomechanical limits (International Organization for Standardization, 2016).

Body part	K (N/mm)	C (shore)	$F_{\max}\;\left(N\right)$
Skull and forehead	150	A 70	260
Face	75	A 70	130
Neck	50	A 70	300
Back and shoulders	35	A 30	420
Chest	25	A 70	280
Abdomen	10	A 10	220
Pelvis	25	A 70	360
Upper arms and elbow joints	30	A 30	300
Lower arms and wrist joints	40	A 70	320
Hands and fingers	75	A 70	280
Thighs and knees	50	A 30	440
Lower legs	60	A 30	260

### 5.2 Finetuning the control parameters

In order to validate the parameters of the gravity and friction models as well as the stability and performance of the control scheme, an open-loop test is performed. For the experiment, the reference of the input signal u is set to zero: $\bar{u}=0$ . The link is then released at an angle of 90° with zero initial velocity. Due to the gravity and friction cancellation and according to Equation 23 one expects the robot to oscillate forever with a natural frequency equal to:

$$\hat{T}=2\pi \sqrt{\frac{J_{d}}{K_{d}}}.$$ (24)

The parameters must be tuned in such a way that the measured oscillations coincide with the theoretical period calculated with this equation. An example with virtual parameters $J_{d}≔\hat{J}$ and $K_{d}≔3.2\;Nm$ results in an expected period period of $\hat{T}=3.92\;s$ and resulted in an actual period of T=4.00 s , with a relative error of 2.04% . This identification yields the friction parameters listed in Table 2.

**TABLE 2:** Simulation and experiment parameters.

Gear ratio	i=222
Total load-side reflected inertia	$\hat{J}=1.2445\;kg.m^{2}$
Link length	l=145 mm
PILZ PRMS stiffness	K=10 N/mm (abdomen)
Luenberger gains	$l_{1}=l_{2}=10$
HOSM gains	N=50
	$\alpha_{1}=1.1$ , $\alpha_{2}=1.5$ , $\alpha_{3}=2$
Friction model	$\hat{\nu }=0.1\;\mu Nm.s$
	${\hat{\tau }}_{c}=0.0065\;Nm$
Virtual parameters	$\hat{J}/J_{d}=2$ , $C_{d}=3.4\;Nm.s$
	$K_{d}=5\;Nm$

To validate the limited influence of the friction model parameters on stability and external torque estimation, a virtual test is performed using the VT model. A disturbance torque with frequency 1 Hz and amplitude 100 Nm is applied at the output, while the reference load-side position is fixed at 0° . The virtual parameters are $K_{d}=5\;Nm/rad$ , $C_{d}=3.5\;Nms/rad$ , and $\hat{J}/J_{d}=2$ . Under perfect model assumptions, the system should behave according to Equation 23. The actual disturbance torque $\tau_{d}\left(t\right)$ is compared with its estimate ${\hat{\tau }}_{d}\left(t\right)$ using:

$$error=\frac{\int_{0}^{10}\left|\tau_{d}\left(t\right)-{\hat{\tau }}_{d}\left(t\right)\right|dt}{\int_{0}^{10}\left|\tau_{d}\left(t\right)\right|dt}.$$ (25)

A large viscous mismatch of $\hat{\nu }/\nu =100$ results in a 9.0% deviation and a 5.46% amplitude error. The influence of Coulomb friction is larger: ${\hat{\tau }}_{c}/\tau_{c}=2$ yields a 2.26% error, while ${\hat{\tau }}_{c}/\tau_{c}=5$ gives 9.0% , with a 5.8% amplitude error. These results indicate good robustness to friction model mismatch. Moreover, stability is largely unaffected, as mismatch mainly impacts disturbance estimation while remaining stable over a wide range of Coulomb friction values. However, for $\hat{\nu }/\nu =710$ , the system becomes unstable.

This experiment can also be used to experimentally determine the minimum value the programmer can choose for virtual inertia $J_{d}$ . Due to the closed loop control and filtering of signals, the system might become unstable when the ratio $\hat{J}/J_{d}$ becomes too high. Increasing $\hat{J}/J_{d}$ too much might also result in current saturation, resulting in a truncated torque signal, with instability as a consequence. For the actuator in Figure 5 a ratio of $\hat{J}/J_{d}=3$ is the point before instability.

### 5.3 Torque estimation accuracy

As explained earlier, the main objective is to ensure the safety of high-geared robots during potential human–robot impacts. In this experiment, the robot (see Figure 5) is position-controlled from 180° to 270° and back using a linear-segment-with-parabolic-blends trajectory, resulting in a nearly constant velocity over a defined interval. Since the PRMS is positioned within this range, an impact inevitably occurs. The trajectory is repeated three times, and the disturbance torque is recorded as a function of time.

**FIGURE 7:**
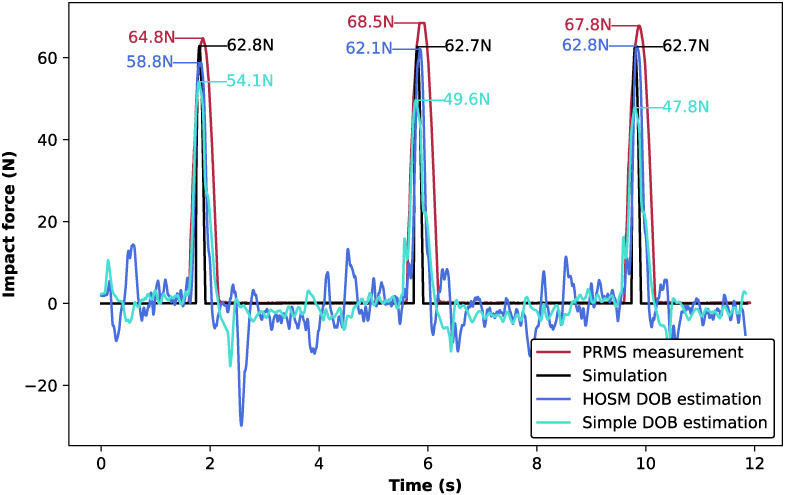
Comparison of instantaneous collision force from PRMS, disturbance observers, and the virtual twin at 2000rpm motor speed. The PRMS setup represents the abdomen with $K_{d}=5Nm$ , $C_{d}=3.4Nm.s$ , and $\hat{J}/J_{d}=2$ . Maximum discrepancies are 8% (sim-to-real) and 8.5% (HOSM DOB vs. PILZ).

The chosen virtual parameters are $K_{d}≔5\;Nm$ , $C_{d}≔3.4\;Nm\;s$ , and $J_{d}≔\hat{J}/2$ . The hardware parameters are listed in Table 2. The experimental results are shown in Figure 7, comparing the HOSM DOB with the simple DOB. Controller parameters (Table 2) are tuned to achieve the fastest possible disturbance estimation while maintaining sufficient stability margins. The first order low pass filter constants for current and motor speed measurements are identical in both experiments.

For the three impacts shown in Figure 7, the peak time of the disturbance estimate is 0.26 s for the HOSM DOB and 0.30 s for the simple DOB. The mean relative error between the estimated and measured impact amplitudes is 8.5% for the HOSM DOB and 21.8% for the simple DOB. While the peak times are similar, the HOSM DOB achieves substantially better amplitude estimation. Considering both peak time and amplitude, the observer performance is quantified as

$$\gamma =1-\frac{{\hat{A}}_{\text{HOSM}}/t_{\text{peak,HOSM}}}{{\hat{A}}_{\text{simple}}/t_{\text{peak,simple}}}=1-\frac{\left(1-\nu_{\text{HOSM}}\right)\;t_{\text{peak,simple}}}{\left(1-\nu_{\text{simple}}\right)\;t_{\text{peak,HOSM}}},$$ (26)

where ν is the relative peak amplitude error, $\hat{A}$ the estimated peak amplitude, and $t_{\text{peak}}$ the half-width of the estimated peak. Using this metric, the HOSM DOB outperforms the simple DOB by 35% in detecting high-frequency impacts.

Additional experiments with the HOSM DOB across different impacted body parts show that peak amplitude estimation errors of 10% or less are achieved for softer impacts (e.g., abdomen, lower legs), while errors increase for more rigid impacts (e.g., skull, forehead), reaching up to 20– 40% in some cases. This is because the impact duration lasts around 0.15s for stiff impacts, which is half the amount for softer impacts. Nevertheless, this reduction in accuracy is acceptable, as safety is maintained as long as the relevant thresholds are respected and the system remains stable.

### 5.4 Virtual twin accuracy

The impact measurements obtained from physical experiments can be compared with the corresponding results from the virtual twin (VT) implementation. The parameter values used in both the simulation and the experimental setup are listed in Table 2. Accurate disturbance estimation is essential for ensuring system stability; therefore, an experiment is conducted to compare the simulated and measured disturbance torques, specifically in terms of estimated impact amplitudes.

The virtual twin employs the same control parameters as the real system, including identical low-pass filters on torque and position with matching time constants. The results are presented in Figure 7, alongside the experimental data from the previous section. The virtual twin exhibits a maximum peak amplitude error of 8% with respect to the real-life measurements, indicating a good level of agreement.

### 5.5 Extrapolation to higher speeds and assessment of ISO/TS 15066 norm

**FIGURE 8:**
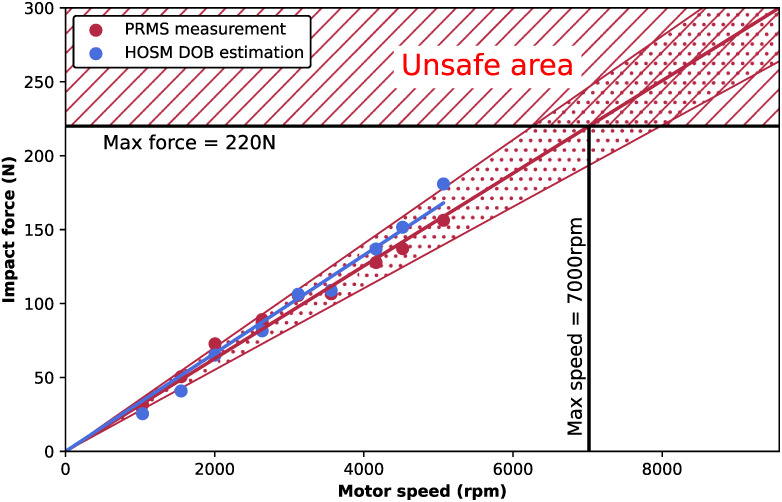
Impact force versus speed for the “abdomen” scenario: measured (PILZ) and estimated using the HOSM DOB. Impedance: $\hat{J}/J_{d}=2$ , $C_{d}=3.2\;Nm\cdot s$ , $K_{d}=5\;Nm$ . ISO/TS 15066 limit: 222 N . Extrapolation predicts a maximum motor-side speed of 7000 rpm within the safe force limit.

The impact experiments in the previous subsections are all performed at the same speed. However, from Equation 3 it is obvious that the relationship between motor speed and impact force is linear:

$$\frac{F_{\text{max}}}{{\dot{\theta }}_{M,\text{max}}}=\frac{l}{i}\sqrt{\mu K},$$ (27)

with l the length of the link. As explained earlier, this formula does not take into account the fact that we can mitigate the impact by means of control. However, we will assume that the impact force is linear with respect to the impact speed. An experiment of ‘abdomen’ impacts versus speed is recorded in Figure 8. The 90 % confidence interval is also added for the measured data points. The linear trend implies that the interpretation of the previous results can be extrapolated to higher speeds. In that figure, an extrapolation is also shown by means of a trend line. Table 1 states that the maximum allowable transient impact force for this collision scenario is 220 N . By extrapolating the measurements, the maximum allowable speed for the actuator of Figure 5, control scheme of Figure 4 and collision scenario ‘abdomen’ is identified at 7000 rpm motor speed.

To ensure that the actuator operates safely, the maximum allowable speed for all collision scenarios listed in Table 1 must be determined. The smallest of these values is then selected as the maximum permissible operating speed in accordance with the ISO/TS standard. Notice that this threshold does not necessarily correspond to the one calculated with Equation 3 because it does not take into account control choice, as explained earlier. Most literature only takes the collision scenario with the lowest biomechanical limit to be the worst collision scenario and determines the maximum speed from that scenario (Fischer et al., 2023). However, it is proven in (Fischer et al., 2023) that parameters like robot hardness and orientation have a significant influence on impact force and pressure.

Another influencing factor is the non-optimized actuator used in the experiments presented in this work. Due to the high gear ratio of 222:1 and the formulation of the total load-side reflected inertia in Equation 17, it follows that the reflected inertia is strongly dominated by the motor inertia $J_{M}$ . Consequently, an optimized actuator design should aim to reduce this contribution by selecting an appropriate motor. A reduction in reflected inertia is furthermore beneficial from a control perspective, as it allows for a lower virtual inertia without inducing instability, ultimately resulting in improved impact mitigation and a safer actuator. The influence of the load-side inertia on the total reflected inertia can be quantified through the sensitivity $S_{J_{L}}=\frac{J_{L}}{J}\frac{\partial J}{\partial J_{L}}=\frac{J_{L}}{J_{L}+i^{2}J_{M}}\overset{i\to \infty }{\to }0$ . For the actuator under consideration (Table 2), this sensitivity equals 2.50 % , indicating a limited contribution of the load-side inertia. This observation supports the feasibility of extending the approach to multi-degree-of-freedom (MDOF) robotic systems. Although the load inertia in such systems varies with configuration, its influence on the joint-level reflected inertia remains small, thereby preserving the effectiveness of the proposed methodology.

Nevertheless, additional experimental studies are required to evaluate the impact of these design improvements on collision performance. Assuming a maximum allowable motor speed of 7000 rpm (corresponding to 32 rpm at the load side for a 222:1 gear ratio), a comparison can be made with existing collaborative robots. The maximum base-joint speed of the UR3e, UR5e, and UR10e is approximately 30 rpm ^4^, and these systems are commonly reported to employ harmonic-drive transmissions^5^. The lower bound of the 90% confidence interval at 7000 rpm corresponds with 28 rpm , which is only 6.5% lower than the maximum speed of 30 rpm . In this context, the Wolfrom-based actuator emerges as a promising alternative to conventional harmonic-drive solutions. Moreover, its inherent backdrivability makes it a viable alternative to quasi-direct-drive actuators, offering a favorable compromise between safety, performance, and mechanical complexity.

## 6 Conclusion

This work presented a torque-sensorless control approach for a high-ratio, backdrivable Wolfrom-based actuator intended for safe physical human–robot interaction.

By deriving a complete analytical model of the actuator and establishing an expression linking gear ratio and inertia ratio to actuator bandwidth, this paper provided theoretical grounding for the claim that high-ratio and inertia reduces the bandwidth of rigid actuators. To address the absence of dedicated torque sensing hardware, a higher-order sliding-mode disturbance observer was designed to estimate external torques using only motor-side sensing. Integrated within an impedance control framework, this allows active shaping of the reflected inertia and reduces collision forces during contact with humans.

The proposed control architecture was validated experimentally using a single-degree-of-freedom robotic setup performing impacts against a Pilz Robot Measurement System. Results demonstrate that, for an actuator incorporating a 222:1 transmission ratio, the observer-based control design can reduce the perceived inertia by up to 1/3 and in this way estimate the transient impact torques with an accuracy below 10% for soft-body collision conditions while maintaining compliance with the ISO/TS 15066 safety limits at significant cobot speed.

Overall, the findings indicate that Wolfrom-based gearing offers a compelling alternative to harmonic-drive and quasi-direct-drive actuation in human-centered robotics by achieving high torque density, compactness, and intrinsic backdrivability. The presented disturbance observer-based impedance control strategy further enhances safety and interaction quality, suggesting strong potential for deployment in collaborative manipulators, humanoids, and wearable robotic systems.

In future work, a more refined mathematical formulation of backdrivability will be developed to support both actuator design and control, thereby contributing to the development of both safe and performative robotic systems. In addition, a more comprehensive model will be proposed that integrates actuator dynamics more rigorously, such as gearbox efficiency and load dependent friction.

## Data Availability

The raw data supporting the conclusions of this article will be made available by the authors, without undue reservation.

## Footnotes

1
https://ailos-robotics.com/

2
https://www.pilz.com/en-INT/products/robotics/prms/prms

3
https://www.pilz.com/eshop/Robotics/PRMS-Collision-measuring-set-for-HRC/PRMS-Collision-measuring-set-for-HRC/Accessories-PRMS/PRMS-compress-kit/p/9A000017

4
https://www.universal-robots.com/media/1802432/e-series-brochure.pdf

5
https://youtu.be/oje2ucwe3vI?si=VHhPOknyv9P920KU
